# Supplementary material for: T-cell commitment inheritance—an agent-based multi-scale model
Source: NPJ Syst Biol Appl. 2024 Apr 17;10:40. doi: 10.1038/s41540-024-00368-y (PMC11024127; doi:10.1038/s41540-024-00368-y)
Supplement: Supplementary file 1 — Supplementary Material [file 41540_2024_368_MOESM1_ESM.pdf]

Supplementary information to

T-cell commitment inheritance – an agent-based multi-scale model

Emil Andersson, Ellen V. Rothenberg, Carsten Peterson & Victor Olariu

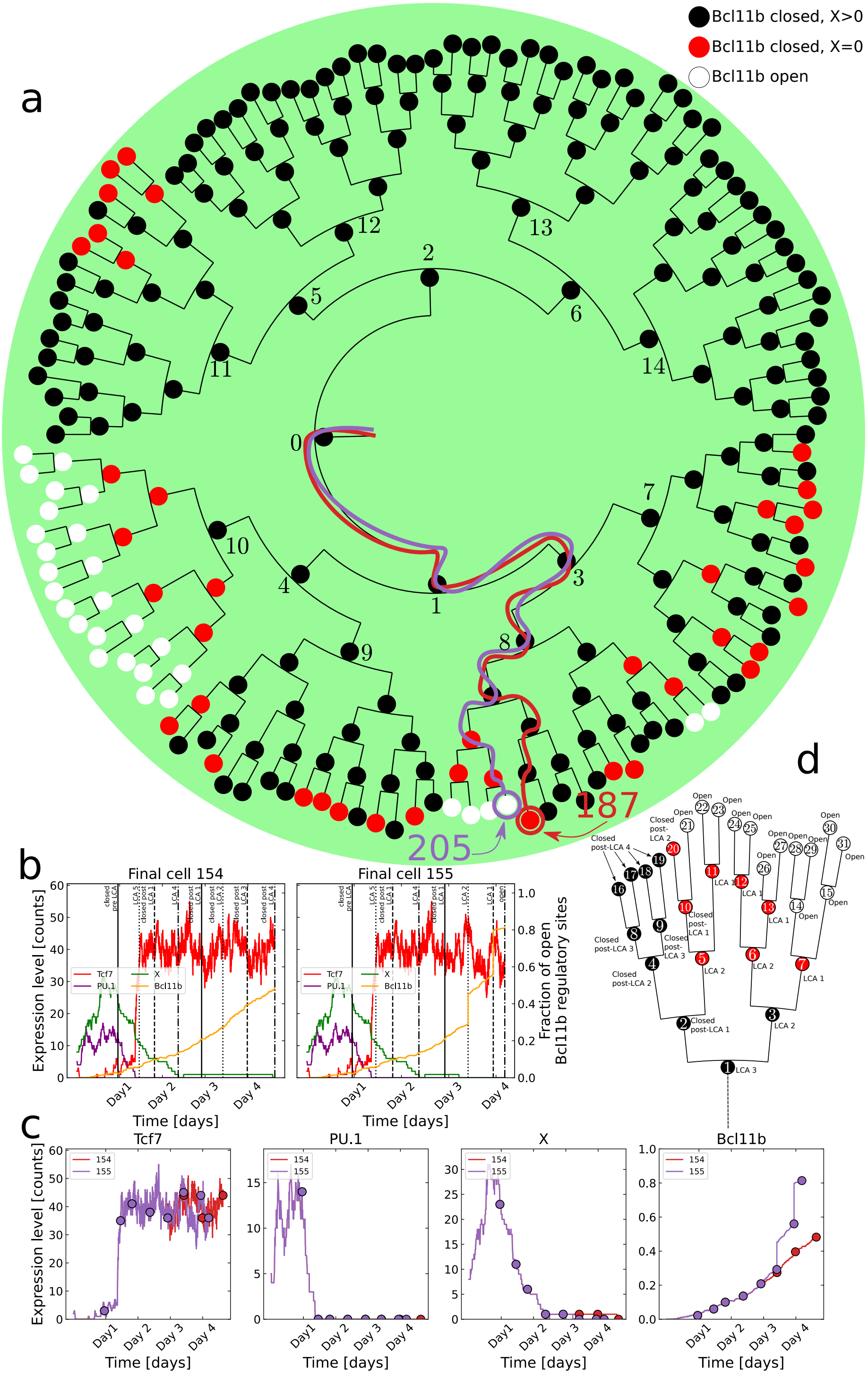

Supplementary Figure 1: Stochastic simulations of cell lineages.

a) The same lineage tree as in Fig. 3. Two additional cell lineages are marked with red and purple lineage paths respectively. Black nodes depict cells with the Bcl11b regulatory region closed and X expression greater than 0, red nodes represent cells where the Bcl11b is closed and X is depleted, and white nodes represent cells where the Bcl11b region is open.

b) Each panel shows the gene expression dynamics for each of the two marked lineages respectively. The vertical lines represent cell divisions and are labelled with the cells' corresponding last common ancestor (LCA) categories.

c) Each panel show the expression for Tcf7, PU.1, X and the fraction of open Bcl11b regulatory sites respectively for the two marked cell lineages. The coloured dots indicate cell divisions.

(d) Synthetic example branch illustrating 'Closed post-LCA' cells. Cell 1 is an 'LCA 3' and its daughter branch rooter at cell 3 is opening up Bcl11b in all cells in the final generation. The daughter branch rooted at cell 2 has two fates. The granddaughter branch rooted at cell 4 stays 'Closed post-LCA' where the order increases by one for each generation. The granddaughter branch rooted at cell 5 becomes LCA again with open descendants.

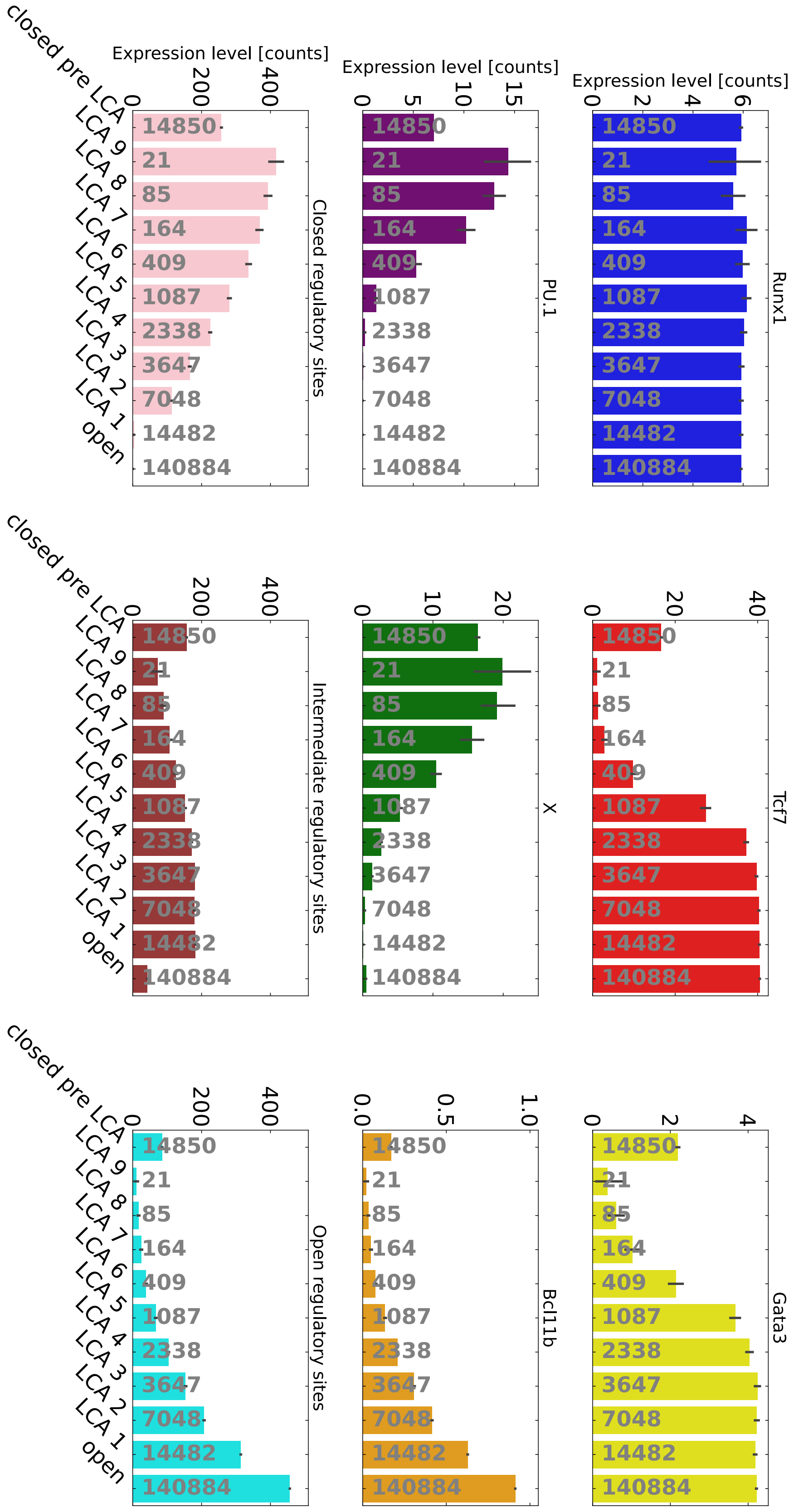

Supplementary Figure 2: Last common ancestor (LCA) statistics.
Mean expression level for all simulated genes and number or regulatory site statuses for cells belonging to the different LCA categories. The categories are ordered in an approximate developmental order. The grey numbers indicate the number of cells belonging to each category. The coloured arrows highlight interesting events described in the main text. The error bars represent standard deviations.

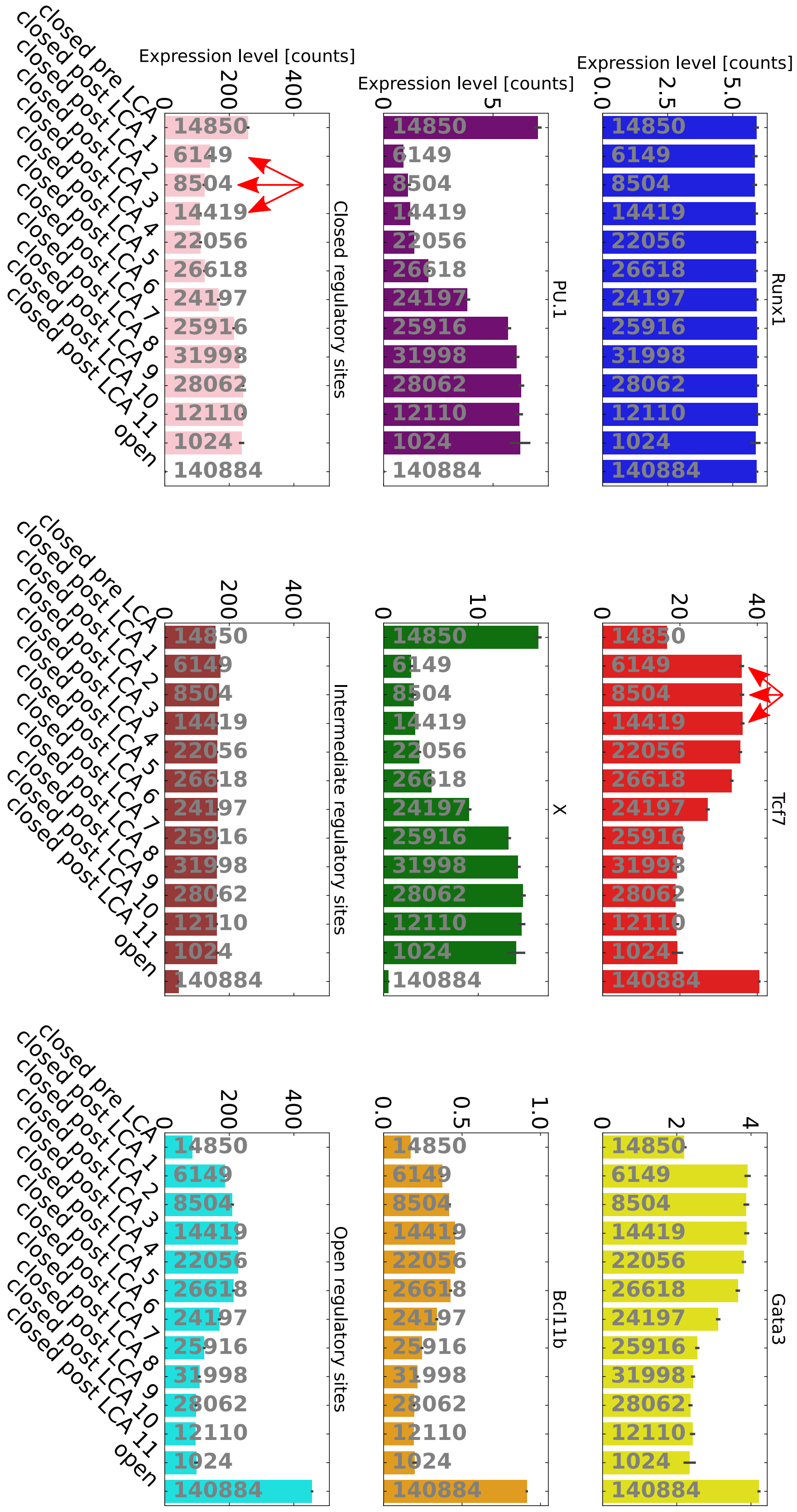

Supplementary Figure 3: Last common ancestor (LCA) statistics.
Mean expression level for all simulated genes and number or regulatory site statuses for cells belonging to the different 'Closed post-LCA' categories. The categories are ordered in an approximate developmental order. The grey numbers indicate the number of cells belonging to each category. The coloured arrows highlight interesting events described in the main text. The error bars represent standard deviations.

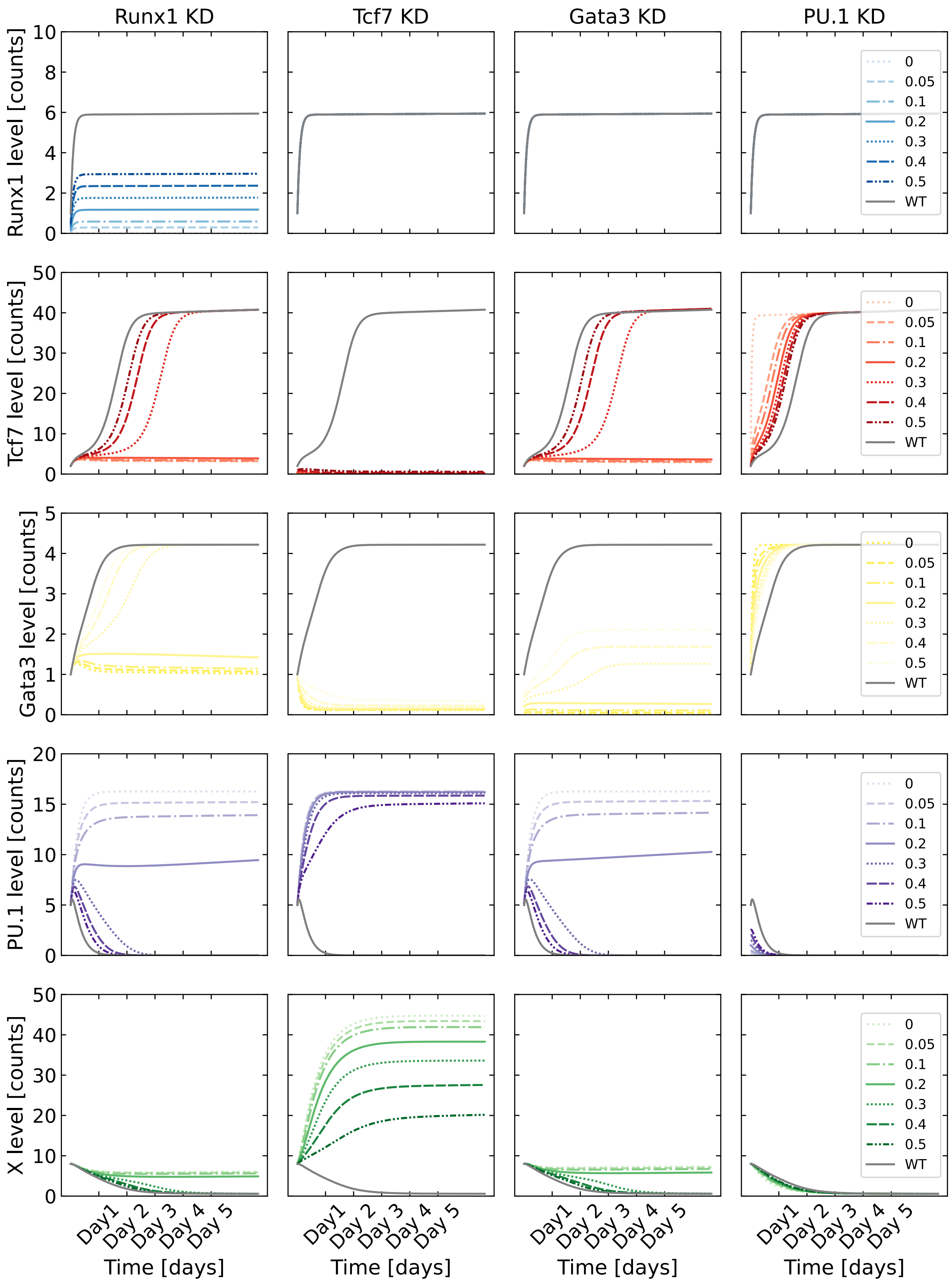

Supplementary Figure 4: Deterministic knockdown simulations.
Each row shows a deterministically simulated gene where a different gene has been knocked down in each column. The knockdown fraction vary between 5 % and 50 % and is denoted by different line stiles and colour shades. The solid grey line represent wild-type simulations.

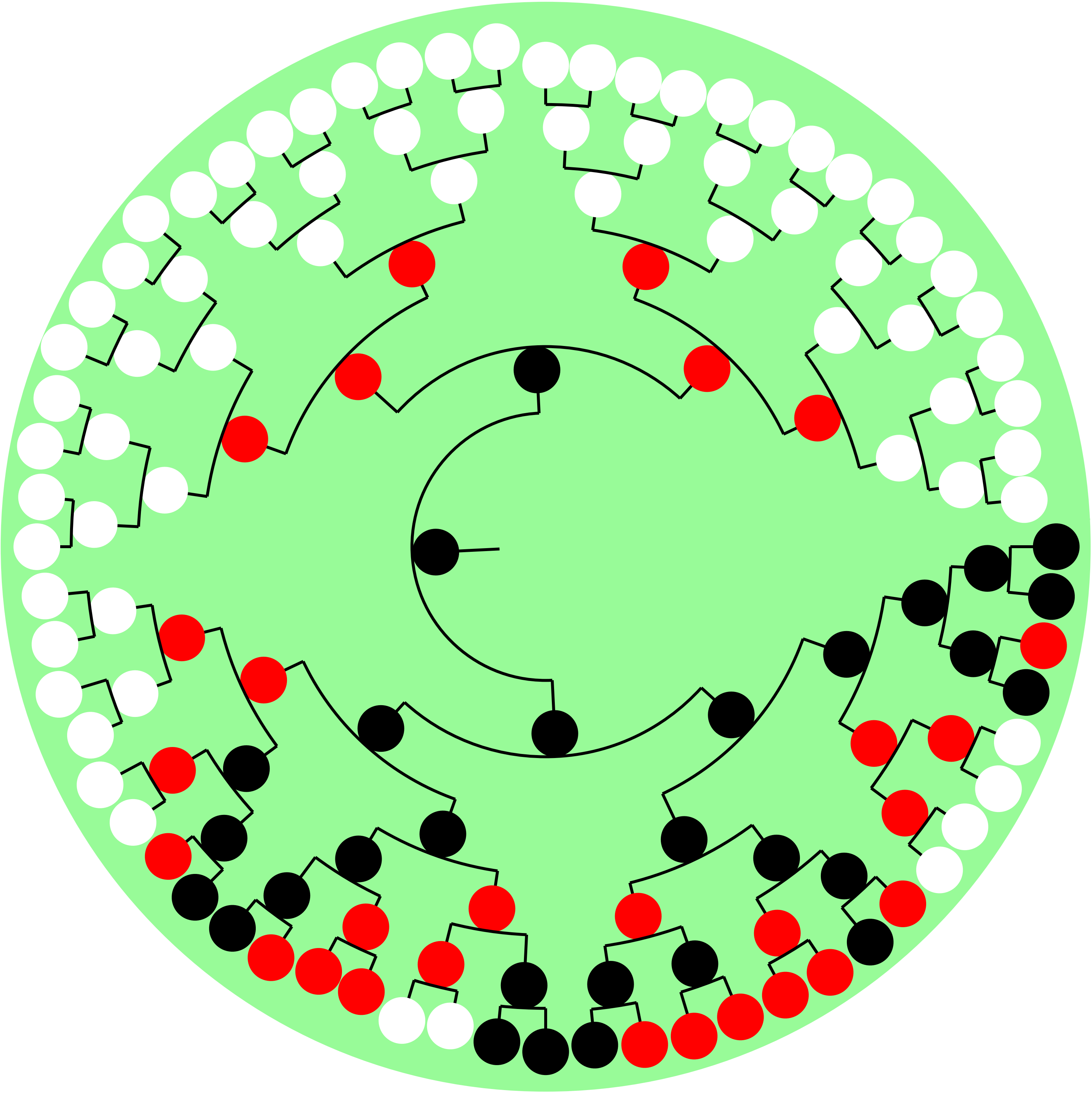

Supplementary Figure 5: Example lineage tree of simulated T-cell colony with 6 divisions.

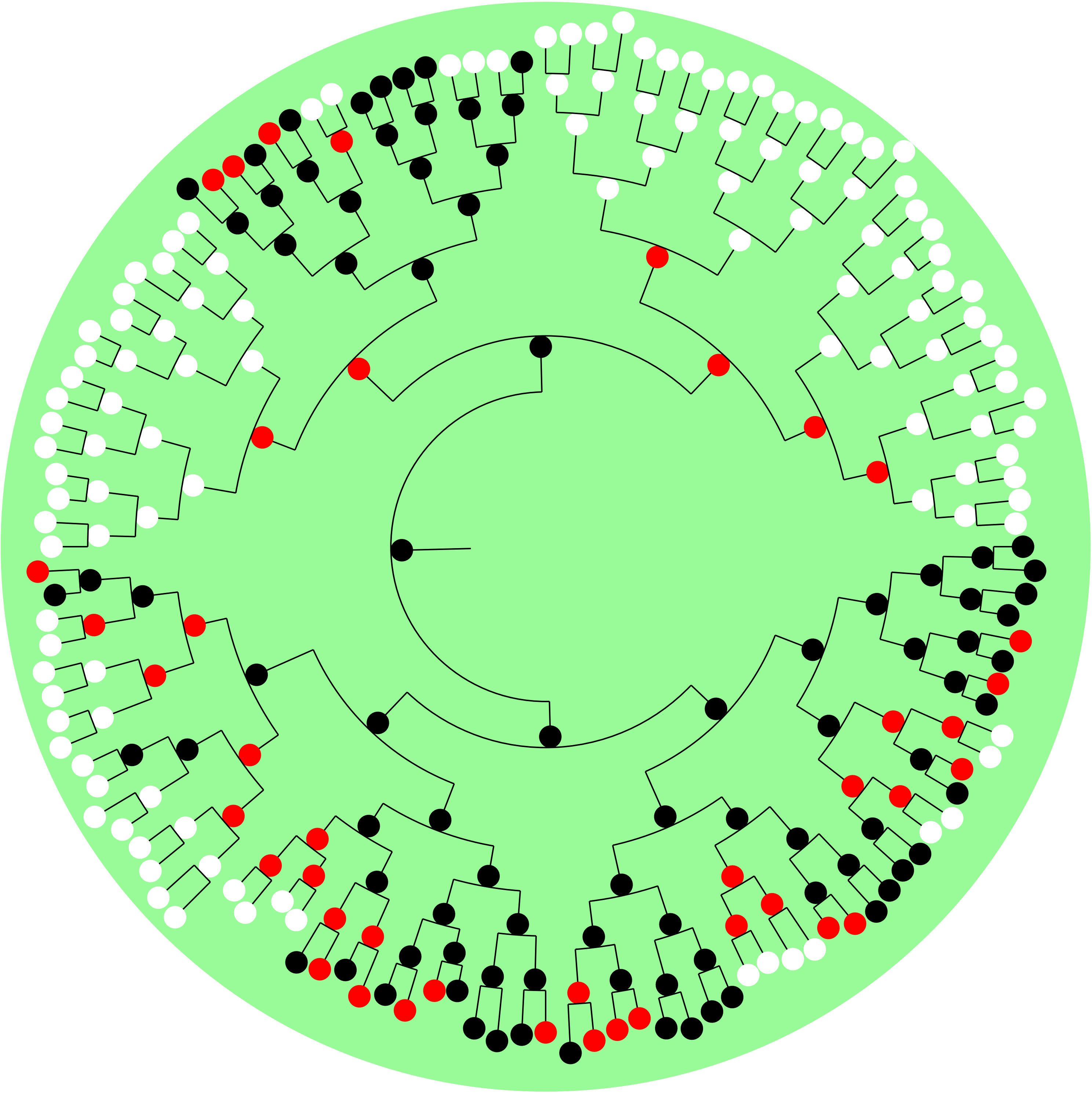

Supplementary Figure 6: Example lineage tree of simulated T-cell colony with 7 divisions.

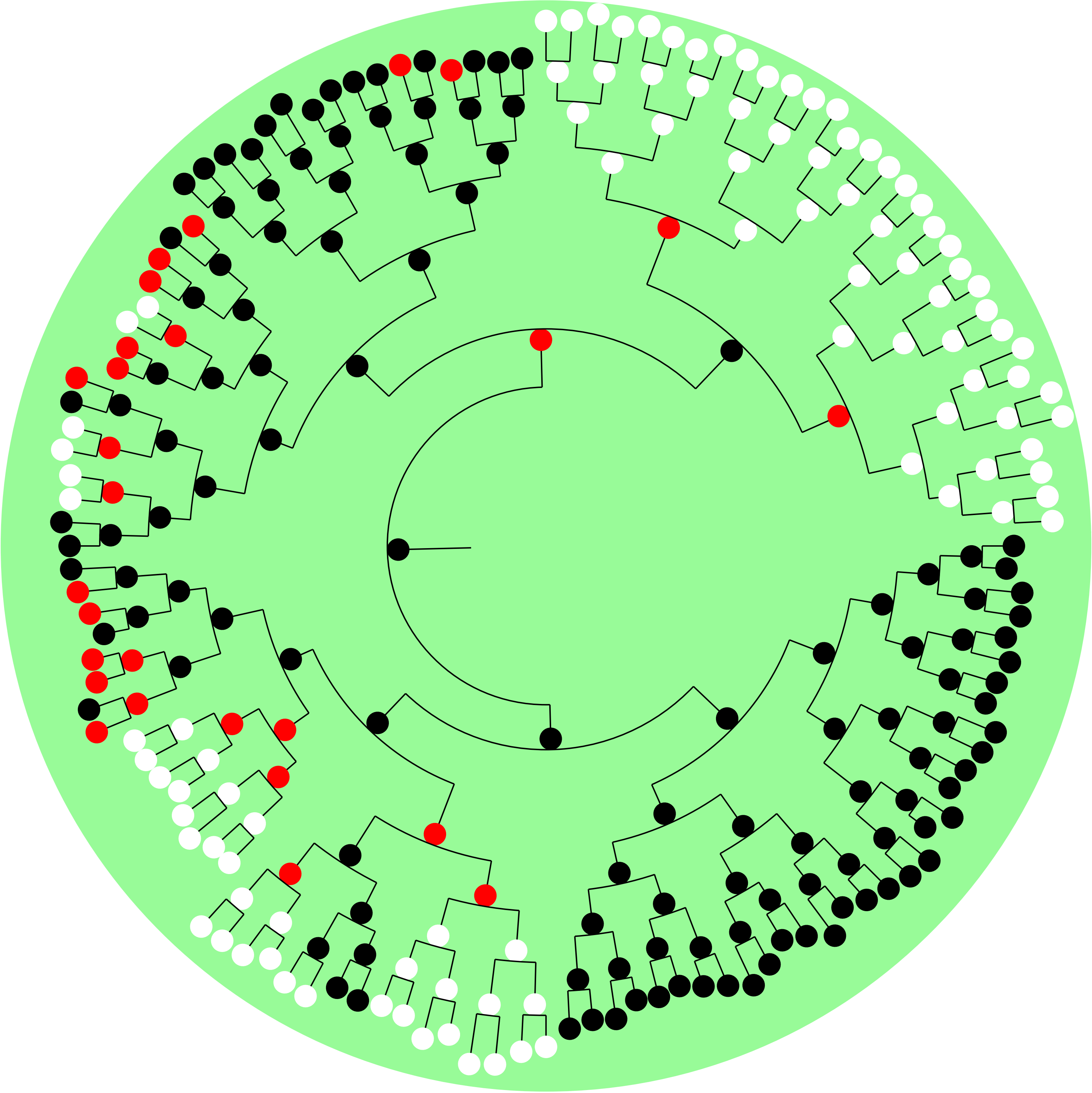

Supplementary Figure 7: Example lineage tree of simulated T-cell colony with 7 divisions.

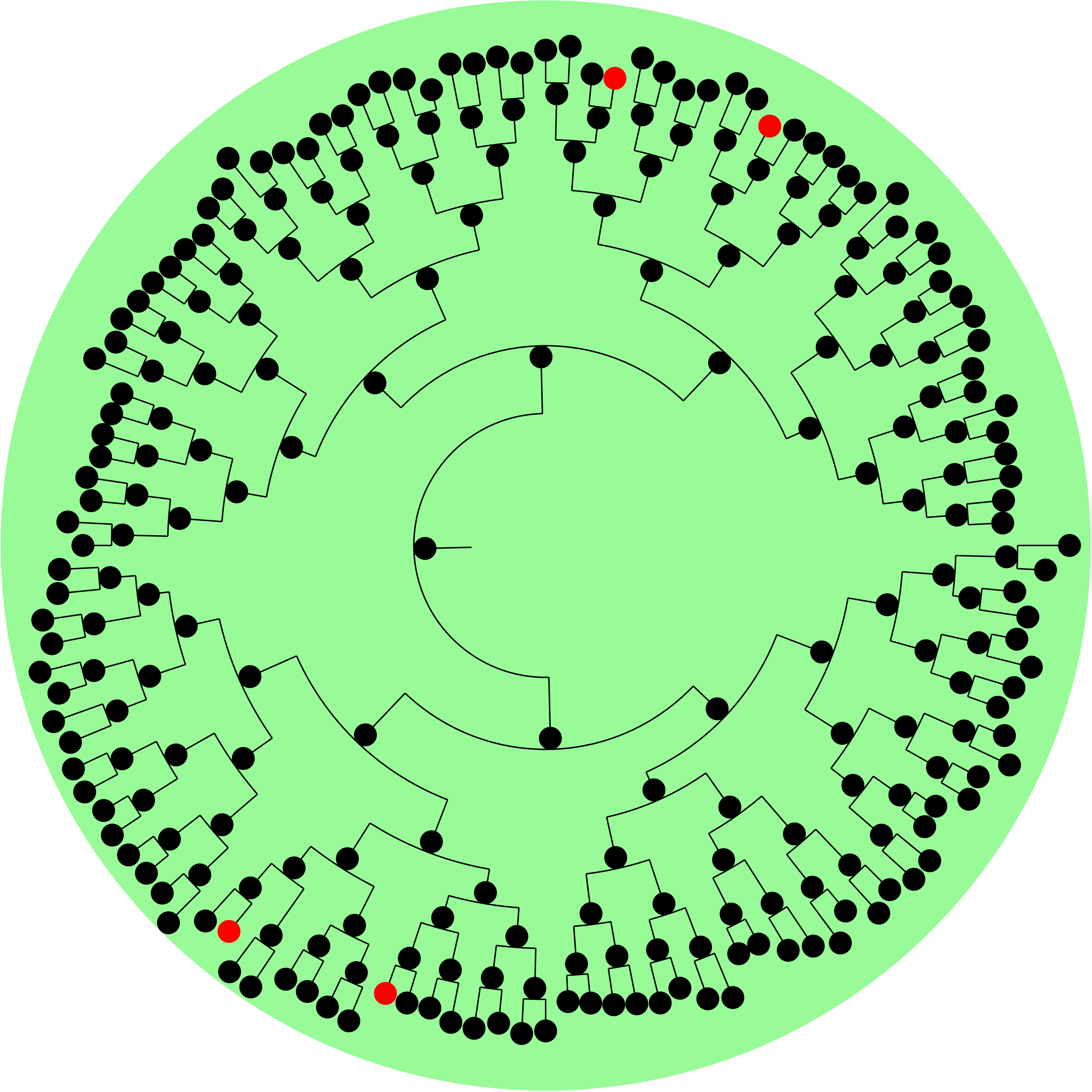

Supplementary Figure 8: Example lineage tree of simulated T-cell colony with 7 divisions.

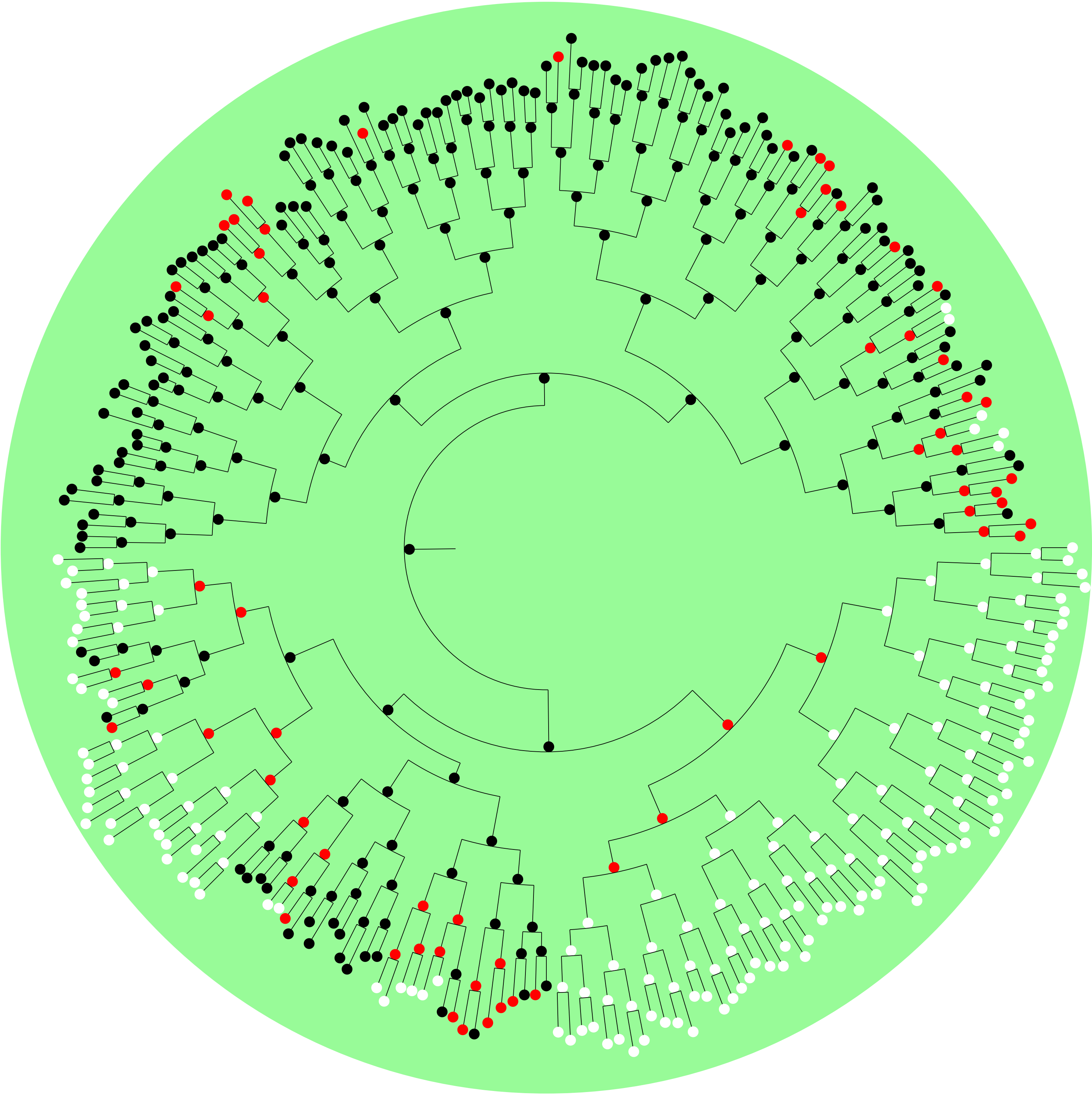

Supplementary Figure 9: Example lineage tree of simulated T-cell colony with 8 divisions.

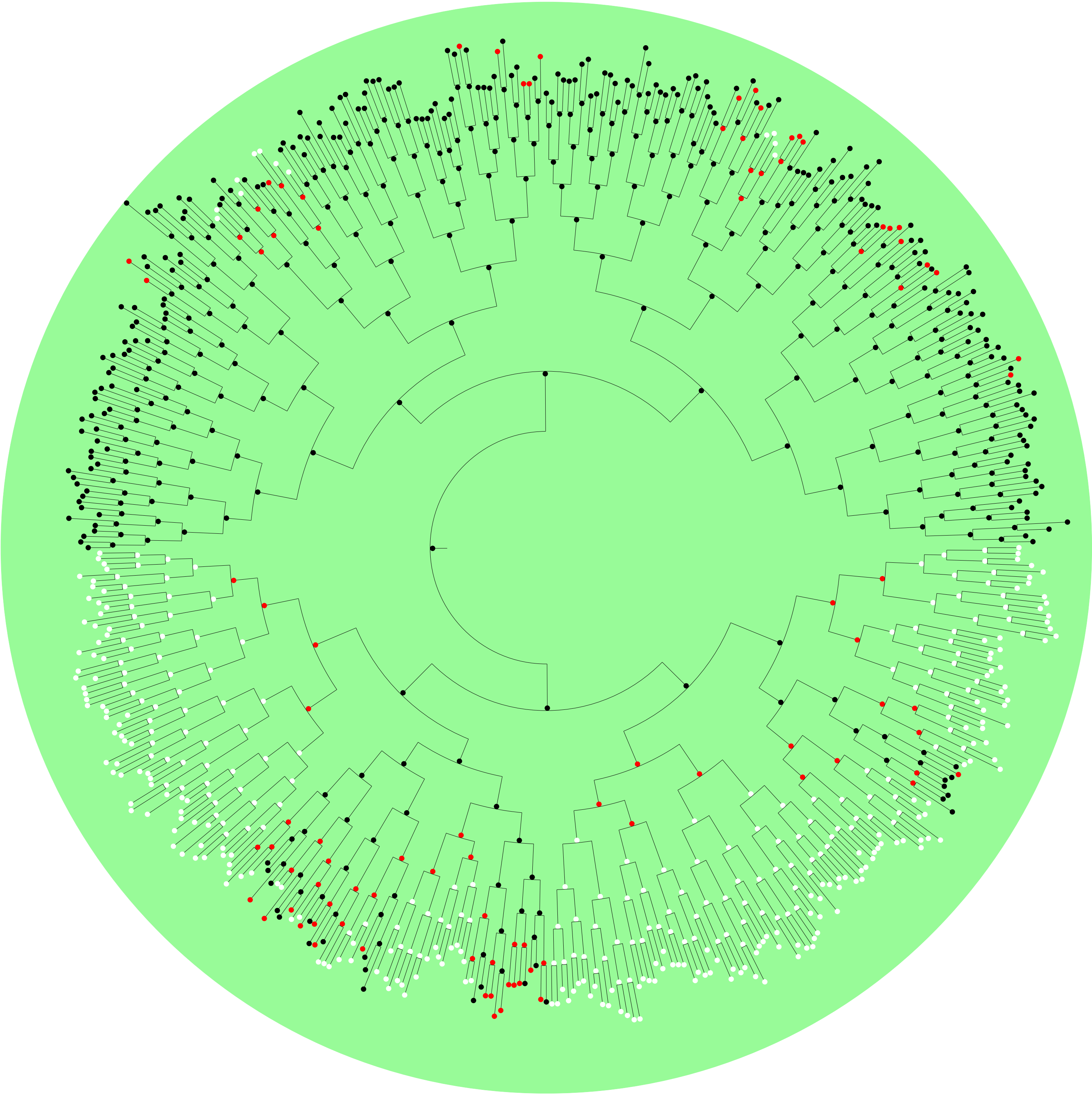

Supplementary Figure 10: Example lineage tree of simulated T-cell colony with 9 divisions.
